# Short-Term Thermal Effect of Continuous Ultrasound from 3 MHz to 1 and 0.5 W/cm^2^ Applied to Gastrocnemius Muscle

**DOI:** 10.3390/diagnostics13162644

**Published:** 2023-08-10

**Authors:** Arely G. Morales-Hernandez, Violeta Martinez-Aguilar, Teresa M. Chavez-Gonzalez, Julio C. Mendez-Avila, Judith V. Frias-Becerril, Luis A. Morales-Hernandez, Irving A. Cruz-Albarran

**Affiliations:** 1 Faculty of Nursing, Autonomous University of Queretaro, Queretaro 76010, Mexico; 2 Education, Movement and Health, Faculty of Nursing, Autonomous University of Queretaro, Queretaro 76010, Mexico; 3 Faculty of Nursing, Autonomous University of Queretaro, Campus Corregidora, Queretaro 76912, Mexico; 4 Laboratory of Artificial Vision and Thermography/Mechatronics, Faculty of Engineering, Autonomous University of Queretaro, Campus San Juan del Rio, San Juan del Río 76807, Mexico; 5 Artificial Intelligence Systems Applied to Biomedical and Mechanical Models, Faculty of Engineering, Autonomus University of Queretaro, Campus San Juan del Rio, San Juan del Rio 76807, Mexico

**Keywords:** continuous ultrasound, infrared thermography, muscle, thermal imaging, medical imaging

## Abstract

Continuous ultrasound is recognized for its thermal effect and use in the tissue repair process. However, there is controversy about its dosage and efficacy. This study used infrared thermography, a non-invasive technique, to measure the short-term thermal effect of 3 MHz continuous ultrasound vs. a placebo, referencing the intensity applied. It was a single-blind, randomized clinical trial of 60 healthy volunteers (19–24 years old) divided into three equal groups. Group 1:1 W/cm^2^ for 5 min; Group 2: 0.5 W/cm^2^ for 10 min; and Group 3: the placebo for 5 min. The temperature was recorded through five thermographic images per patient: pre- and post-application, 5, 10, and 15 min later. After statistical analysis, a more significant decrease in temperature (p<0.05 ) was observed in the placebo group compared with the remaining groups after the application of continuous ultrasound. Group 1 was the one that generated the highest significant thermal effect (p<0.001), with an increase of 3.05 °C at 15 min, compared with the other two groups. It is concluded that to generate a thermal effect in the muscle, intensities of ≥1 W/cm^2^ are required, since the dosage maintained a temperature increase for more than 5 min.

## 1. Introduction

Continuous ultrasound (CUS) is considered a deep-penetrating agent consisting of sound waves that transmit energy to achieve a therapeutic effect [1]. In this way, it interacts with the body tissues, causing vibrations that result in a thermal effect [2]. It is used in physiotherapy to treat musculoskeletal-type injuries and other soft tissues [3,4]. Several articles discuss its effectiveness in reducing pain and improving functionality after its application [5,6,7,8,9,10]. It has even been compared with the placebo effect, obtaining significant differences in favor of therapeutic ultrasound [11,12,13]. Among its beneficial effects are the change in membrane permeability, the promotion of connective tissue extensibility, the modification of nerve conduction, and the increase in temperature in the tissue [1]. These effects intervene in the tissue repair processes, generating specific benefits such as pain control and reduction, improved range of motion, and greater exercise tolerance, impacting the patient’s functional capacity and recovery [3,4,5,6,7,8].

Regarding the measurement of the increase in temperature in the tissues, intramuscular thermometers or thermocouples were the tools used to quantify its increment. However, they had the risk of generating injury or infection in the patient [14,15,16,17,18]. Therefore, the need arose to implement new tools that measure the thermal effect generated by the CUS and thus substantiate the guidelines used in the treatment. Infrared thermography (IT) is a technique that measures the radiated heat of a body through an infrared camera [19]. The camera is responsible for receiving and quantifying the radiation emitted by an object, which is displayed as a thermal image where the color of each pixel is associated with a temperature [20]. Thermographic cameras can detect temperature increases that occur in anatomical areas without needing physical contact with the object, thus monitoring acute or chronic injuries at the muscular, bone, tendon, and joint levels [21,22]. Thus, the use of infrared thermography in clinical trials has the advantage of protecting the patient, as it is a non-invasive, safe, and fast tool compared with intramuscular thermometers. IT in physiotherapy has been used to evaluate thermal effects in some treatments, for example, cryotherapy, laser therapy, electrotherapy, diathermy, and massage [23], as it is considered a viable analysis tool. Research focusing specifically on infrared thermography and ultrasound has been developed. For example, Ratajczak et al. [24] carried out a thermal evaluation of the left quadriceps femoris muscle after the application of continuous ultrasound at 0.5 W/cm^2^ intensity and 1 MHz frequency, comparing the application medium used (paraffin oil and gel). The results show an increase in temperature after applying ultrasound using paraffin oil, while gel use shows a decrease in temperature. Similarly, Ratajczak and Boerner [25] evaluated thermal differences through thermography in the Achilles tendon after ultrasound application at 1 and 3 MHz. They used paraffin oil as a coupling medium, and their results show that there is a temperature increase with ultrasound applied at 3 MHz. Boerner and Podbielska [26] measured the thermal impact of two therapies (local cryotherapy and ultrasound) on the anterior surface of the right thigh. Both treatments were applied to the right quadriceps in a different order. Ultrasound was applied at 1 MHz and 0.6 W/cm^2^. The results showed a more significant temperature decrease after the initial application of ultrasound, followed by cryotherapy. Thus, studies show that IT stands out as a tool for the diagnosis, assessment, and follow-up of patients’ physical and muscular conditions [27,28]; in addition, it allows for the evaluation of the efficacy of some treatment techniques used in physiotherapy [29,30]. Based on those mentioned earlier, at present, within clinical practice, there is little research on an adequate standardization of dosage for tissue recovery and the real effects of US [12,31], such as the intensity at which it should be applied.

The aim of this research was to determine the short-term thermal effect of CUS applied to the gastrocnemius muscle through infrared thermography, taking as a reference the intensity applied, to know its efficacy and improve decision making on its use, accuracy, and effects. In this way, this study will contribute to the development of evidence-based physiotherapy by promoting the appropriate choice of physical agents, such as Therapeutic ultrasound, based on scientific knowledge and clinical reasoning. Three groups received different dosages of CUS. These were Group 1 (G1) at 3 MHz with an intensity of 1 W/cm^2^ for 5 min; Group 2 (G2) at 3 MHz with an intensity of 0.5 W/cm^2^ for 10 min; and finally, Group 3 (G3) with a CUS placebo for 5 min. The hypothesis proposed was that an increase in temperature in each group would be more significant in G1 from 3 MHz to 1 W/cm^2^ and that the temperature increase would be maintained for ≥5 min in G1 and G2 to generate a therapeutic effect.

## 2. Materials and Methods

**Figure 1:**
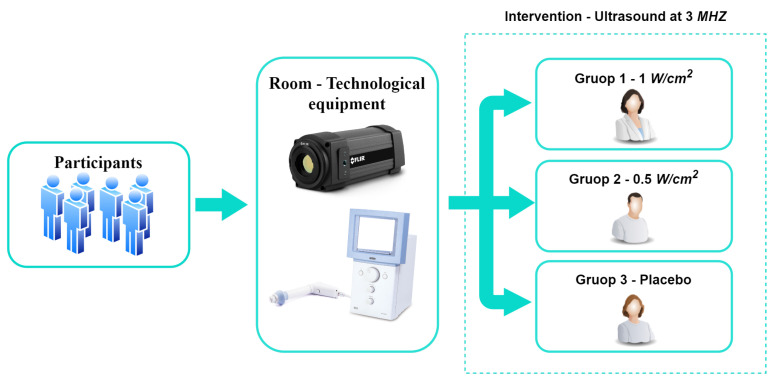
Proposed general methodology.

The general methodology proposed for the development of this study is presented in Figure 1. It shows the participants, the materials used, and the conditions of the intervention.

### 2.1. Participants

The design and type of study was an experimental single-blind randomized clinical trial. The invitation to participate was open. Those who participated signed a letter of informed consent and were granted a letter of confidentiality. The study gathered 60 participants, including 30 men and 30 women; each received a folio as they joined the study. Subsequently, a simple random sampling was performed, dividing each group into three paired groups (*n* = 20) according to sex (10 women and 10 men). G1 had an average age of 21.4 ± 1.3 years, G2’s was 22.3 ± 1.6, and G3’s was 20.6 ± 1.1. Each group was assigned a specific dosage to compare the effects of each. Participants did not know to which group they had been assigned. 

The selection of participants was established through the following criteria. The inclusion criteria were that the participants voluntarily participated in the study with prior informed consent and that they were between 19 and 24 years of age. The exclusion criteria were the presence of thrombophlebitis or hemodynamic alterations; the presence of musculoskeletal trauma in the lower limbs in the last six months; physical activity or sport related to constant trauma in the lower limb; alteration of sensibility in the lower limb; open wounds, skin rash, inflammation, or infection in the lower limbs; diagnosis of an autoimmune disease; or sensitivity to therapeutic ultrasound therapy. Finally, the elimination criteria were that participants did not complete the measurements or that they wished to leave the study. The specific criteria for the use of thermography were the following: comfortable clothing and shorts; not using any type of substance or cosmetic on the skin of the area to be treated; no physical activity on the day of the application; not consuming alcohol or tobacco during the last 24 h; not consuming stimulant drinks; not presenting lesions, allergies, or rashes on the area to be treated; and not presenting any alteration that could modify their body temperature. 

This study was approved by the Research and Bioethics Committee of the Faculty of Nursing of the Autonomous University of Queretaro with registration code FEN_FIS_2020_80, in accordance with the Nuremberg Code (paragraphs 1–10), the Declaration of Helsinki (principles 1.1, 1.12, III, and I), and the Mexican General Health Law (Art. 13, 14, 17, and 100).

### 2.2. Room—Technological Equipment 

**Figure 2:**
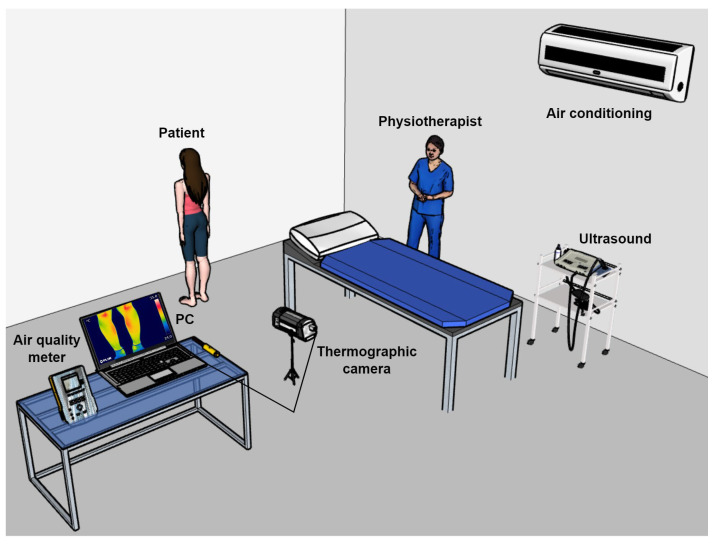
Conditioning room where the intervention took place.

The following technological equipment was used for the investigation: (1) therapeutic ultrasound BTL-5000 SWT POWER; (2) FLIR A310 camera, with a resolution of 320 × 240 pixels and a thermal sensitivity of 0.05 to 30 °C, placed at 1.2 m from the lens; (3) Fluke-61 infrared thermometer; (4) Fluke-975 Air Quality Meter; (5) air conditioning; (6) metronome. The camera software was set with an ambient temperature of 20 °C ± 2 °C and a relative humidity between 40 and 60% (both determined with an air quality meter). The room layout is shown in Figure 2.

### 2.3. Pilot Test

A pilot test was conducted with eight participants who met the selection criteria. However, we tried to ensure that they did not belong to the study population. They were asked to sign an informed consent form. Subsequently, a pilot test was carried out to minimize extrinsic and intrinsic factors that could cause an indirect temperature variation during the CUS application. Therefore, a rectangular template was initially made with the following measurements: 5 cm wide by 10 cm long, which determined the application area and would be used with the 8 participants. In this way, the same application area was guaranteed for each participant.

Regarding the application of the conductive gel, tests were performed with the conventional application, which consists of filling the entire surface of the head with conductive gel; this application was discarded due to heterogeneity among the evaluators and dispersion of the gel outside the application area. Therefore, to avoid such bias, a syringe was used to dose the amount of gel used, and the tests were initially performed with 5 mL and 2 mL amounts. Finally, it was determined that the 2 mL amount was sufficient to cover the US head and remain in the application zone for 5 and 10 min. Regarding the application technique used, the circular and transverse techniques were considered. However, after testing both techniques, it was determined that the transversal technique was more useful for maintaining the gel in the application area. Finally, in order to prevent generating more friction than necessary, it became essential to determine the speed at which the technique should be employed. Therefore, a metronome was used to provide feedback on the movement of the head and ensure uniformity for all participants during the application process.

### 2.4. Intervention

The study took place in the Preclinical Laboratory of the Bachelor’s Degree in Physiotherapy of the Faculty of Nursing of the Autonomous University of Queretaro. On application day, the fundamental guidelines for using thermography in humans were followed, including an air-conditioned laboratory and the lowest possible incidence of light; the room temperature was maintained at 20 ± 2 °C and the relative humidity between 40 and 60%. The thermographic camera was placed at a distance of 1.2 m, maintaining a right angle with the participant. Regarding the US equipment, the first step was to verify that it was connected to a light regulator to protect the equipment. The equipment was turned on, and the dosing parameters were adjusted according to the group. Participants were divided into three groups. For G1, the established dosage was 1 W/cm^2^ for 5 min. For G2, the dosage was 0.5 W/cm^2^ for 10 min. Finally, for G3, an ultrasound placebo was used for 5 min, i.e., the equipment was kept on but no dosage was selected. The frequency of application was 3 MHz due to its superficial and local vascular effects [25,32]. The head was checked to make sure it was functioning properly and was calibrated.

The clinical trial was conducted in five stages. In stage one, each participant underwent a 15 min conditioning period inside a room. During this time, they were required to remain in a relaxed position without crossing their legs or arms. Afterward, the participants signed an informed consent and received a letter of confidentiality. Subsequently, the area of application of the CUS was determined by marking a template measuring 10 × 5 cm on the gastrocnemius muscle in the medial portion of the left leg. Then, the first thermographic image acquisition was performed. In stage two, each participant was placed on a stretcher, and the application of CUS was started with the corresponding group dosing with 2 mL of gel. A Beats Metronome was used, and upon completion, the second thermographic image was obtained. Subsequently, stages three, four, and five involved capturing thermographic images every 5 min for 15 min.

**Figure 3:**
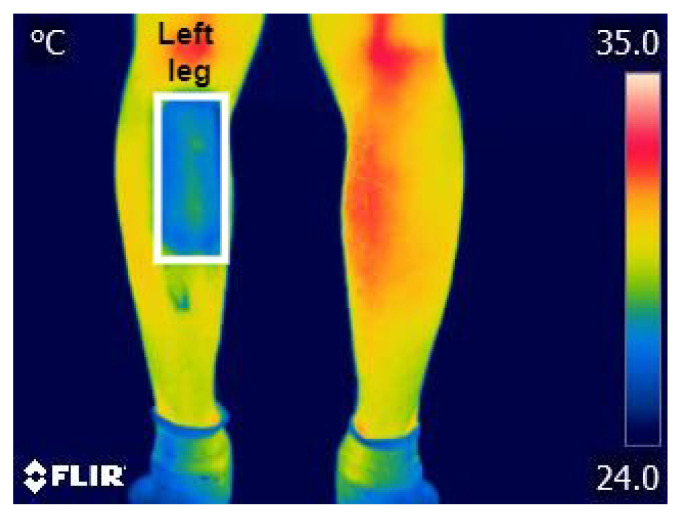
Region of interest analyzed—gastrocnemius muscle.

Thus, 5 thermograms were obtained from each participant, concluding with 300 thermograms corresponding to the 60 participants. Once all the thermograms were obtained, the thermal information from the gastrocnemius muscle of the left leg (LL) was extracted, as shown in Figure 3.

### 2.5. Statistical Analysis

The distribution of the data was evaluated through the Shapiro–Wilk test. Then, to obtain the temperature increase and significance between pre- and post-intervention, a repeated measures ANOVA was performed. Similarly, the effect size was calculated to quantify the magnitude of the differences between the two measurements. Finally, a one-factor ANOVA test with Tukey’s post hoc tests was carried out to evaluate the temperature increase and significance among the three groups for the different stages. The initialization of the analyses was as follows: 20 participants per group (G1, G2, and G3) with their temperature determined for each stage, and for significance, a confidence interval of 95 %. Moreover, Cohen’s effect size (*d*) was classified as small (>0.0 and<0.4), medium (≥0.4 and<0.8), and large (>0.8) [33]. In addition, runtime, memory, sensitivity, and scalability analyses were performed [34,35]. For scalability, two additional statistical analyses were performed, varying the *n* of each group, the first with 16 patients and the second with 18. Similarly, for the sensitivity analysis, the significance threshold was evaluated at 0.05 and 0.10. Finally, it is important to mention that the statistical analysis was performed in SPSS version 25.0.

## 3. Results

The results of the statistical analyses are shown by application group: (1) CUS with a dosage of 1 W/cm^2^ (G1); (2) CUS with a dosage of 0.5 W/cm^2^ (G2); and, finally, (3) the placebo (G3). It is important to note that, with the Shapiro–Wilk test, a normal distribution was observed.

### 3.1. Ultrasound at $1\;W/{cm}^{2}$—Group 1

The results of the data analysis of the regions of interest (ROIs) with the ultrasound with a dosage of 1 W/cm^2^ are shown in Table 1, where the number in front of the “Left Leg” is the stage, $\bar{X}$ is the mean of each ROI, p is the significance, σ is the standard deviation, ∆T is the temperature increase, and d the effect size. After the intervention, the results showed a significant post-application (stage 2) temperature decrease in all the stages. Hence, a more considerable temperature increase occurs at 5 min post-application (stage 3) relative to the remaining 10 minutes (stage 4). At the end of 15 min post-application (stage 5), the temperature did not return to its initial state, remaining below the initial temperature by more than 1 °C. In addition, a large effect size can be observed for all stages.

### 3.2. Ultrasound at $0.5\;W/{cm}^{2}$—Group 2

Table 2 shows the results of the data analysis with the ultrasound at a dosage of 0.5 W/cm^2^. Similarly, a significant post-application (stage 2) temperature decrease was observed in the left leg. In the recovery period, there was a greater increase in temperature in the first five minutes (stage 3) compared with the remaining ten minutes (stage 4). It could be seen that, after 15 min of recovery (stage 5), the temperature did not reach its initial state, remaining below 1 °C; moreover, it can be observed that this temperature decrease is greater than in Group 1. Similarly, a large effect size can be observed.

### 3.3. Placebo—Group 3

Finally, the results of the placebo group are shown in Table 3. A significant decrease in temperature after application (stage 2) is observed. In addition, it can be seen that in stage 3 there was the greatest increase in temperature. Although in the following stages the increase continued, it was smaller. As in the previous groups, the temperature after 15 min remained below the initial temperature (below 1 °C), and it can also be observed that this increase is greater than in the other two groups. Similarly, a large effect size can be observed.

**Figure 4:**
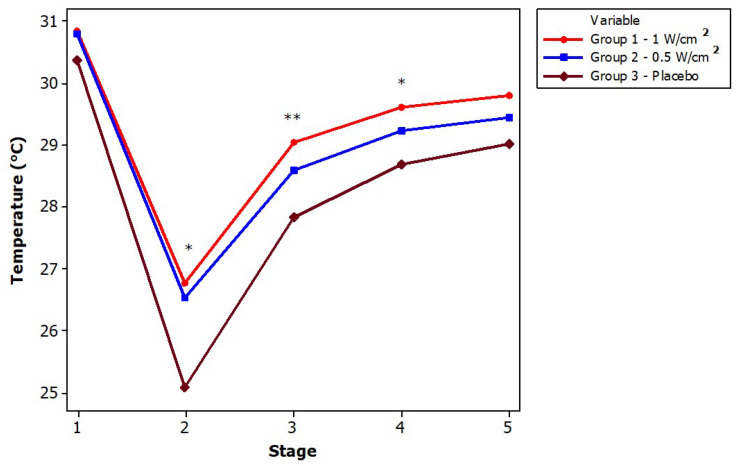
Temperature analysis between groups. Statistical differences between groups are shown with symbols (* p<0.05; ** p<0.01).

Figure 4 shows the statistical analysis of each stage among the three intervention groups. For the first stage, no significant temperature differences were found. In the second stage, there is a significant decrease in the temperature of the placebo group in contrast with the ultrasound at 0.5 W/cm^2^ and 1 W/cm^2^, i.e., the placebo group (G3) showed a greater temperature decrease and a lower final temperature range (stage 5); this showed that the two remaining groups have a different behavior due to the thermal effect of the CUS. For the third and fourth stages, there is only a significant difference in temperature between the placebo group and the application with ultrasound at 1 W/cm^2^, but not for the other cases. Finally, in stage 5 there is no difference between the groups. It is important to note that, although in stage one the temperature of the three groups did not differ significantly, in the following three stages, the behavior follows a trend, with the thermal effect remaining larger in G1 than in the other two groups, while G3 always had a lower temperature than the other two groups.

Regarding the scalability analysis, thermal behavior is still maintained; in terms of *p* values, there are small variations; however, no value changed from significant to non-significant, or the other way around. The runtime of the statistical analysis was 3.4 ± 0.2 s, and the memory consumption was 750 ± 100 M. The more data samples you have, the longer the processing time and the higher the memory consumption. Regarding the sensitivity analysis, some data that previously were not significant are now significant; however, it is important to mention that in general more than 90% of the data were not altered. 

**Figure 5:**
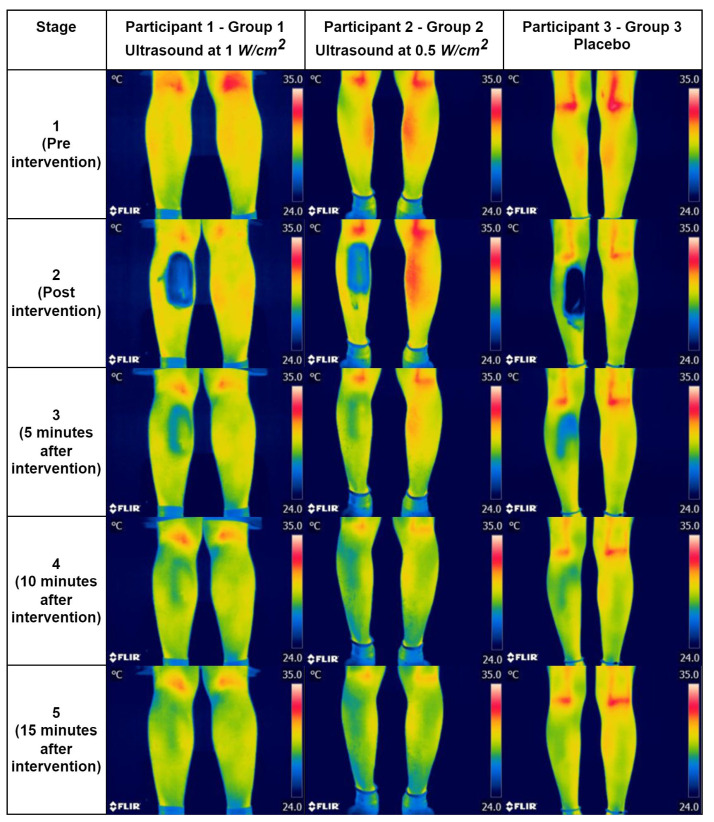
Thermographic images with CUS at 1 W/cm^2^ (Subject 1), at 0.5 W/cm^2^ (Subject 2), and placebo (Subject 3).

Finally, Figure 5 contains examples of the thermographic images showing the qualitative changes by stages in each group (G1, G2, and G3). These images are presented in the rainbow color palette.

## 4. Discussion

The objective of this research was to determine the short-term effect of CUS at 3 MHz with special emphasis on the intensity applied; for this purpose, the CUS was performed at two intensities, 1 W/cm^2^ and 0.5 W/cm^2^, in addition to a placebo. Among the main results, the ANOVA test confirms the thermal effectiveness of CUS (p<0.05), with a greater temperature decrease after the intervention in G3 (placebo) and a greater thermal recovery effect in G1 (1 W/cm**^2^**) after 15 min.

It has been demonstrated that there is an increase in temperature after applying CUS in muscle tissue [15,16,17,36]. An effective conductor is necessary to achieve such an increase, which adequately transmits the sound waves caused by the CUS; Draper [37] mentions that the best conduction medium is water-based gel. However, Ratajczak et al. [24] mention that paraffin oil is a better conductor because it has fewer secondary changes in temperature after CUS application.

Previous studies report that tissue temperature increases linearly after ultrasound application [18]. However, when analyzing the temperature behavior, in this study, a decrease in temperature was observed in the three groups just at the end of the CUS application, as described by authors such as Morishita et al. [14] and Ratajczak et al. [24]. G1 (1 W/cm^2^) and G2 (0.5 W/cm^2^) showed a temperature decrease of 4.10 °C and 4.27 °C, respectively, and for G3 (placebo) a greater decrease was shown with 5.28 °C. Thus, the thermal effect started later than the temperature decrease, as found in the research of Ratajczak et al. [24] and Noble et al. [38]. The decrease in temperature reported could be explained due to the mechanism of thermoregulation by conduction, which corresponds to the transfer of heat between the body and the gel, causing a loss of body heat. This decrease was smaller in G1 (1 W/cm^2^) and G2 (0.5 W/cm^2^) compared with G3 (placebo), due to the thermal effect generated by the CUS. In G3, there was only an automatic loss of body heat due to the contact with the gel.

Lehmann [39] reported the effects obtained after the elevation in tissue temperature, depending on the degrees increased: 1 °C increases the metabolic rate, while elevations of 2 to 3 °C cause pain reduction, muscle spasms, and increased blood flow. Increases of 4 °C or more are necessary to increase collagen extensibility and inhibit sympathetic activity. This study recorded a total temperature increase of 3.05 °C in G1 (1 W/cm^2^), which was the only group that generated statistically significant thermal effects. However, in G2 (0.5 W/cm^2^) there was an increase of 2.93 °C, similar to that reported by Gallo et al. [16], who found as a result that treatment with continuous ultrasound at a dosage of 0.5 W/cm^2^ produces a temperature increase of 2.8 °C ± 0.8 °C above the baseline. The temperature increase observed in G1 (1 W/cm^2^) and G2 (0.5 W/cm^2^) was due to an effect called steady state [18] which is described as a constant state of equilibrium or stationary state, which generates an increase in intramuscular temperature. The treated tissue benefits from the physiological effects of the temperature increase, unlike in G3, where the increase in temperature occurred because of the thermoregulation mechanism.

In this regard, Cameron [1] reported that the minimum intensity to generate a thermal effect is 0.5 W/cm^2^ with an application of 10 min. However, it was demonstrated that the thermal effect of CUS occurs at intensities greater than or equal to 1 W/cm^2^ since the dosing of the two remaining groups did not generate statistically significant changes. This proved that a dosage in less time and with a higher intensity as G1 (1 W/cm^2^) can generate temperature increases equal to or greater than the dosage proposed by Cameron [1].

It has been reported that most of the thermal effects should be maintained for a minimum time of 5 min to be a determining factor in the efficacy of physiotherapy treatments [13]. This research showed that the thermal effect started 5 min after application (stage 3) and was maintained for 10 min (stages 4 and 5) when the therapeutic effects can be exploited.

Thus, Ratajczak et al. [24] made a comparison between the conductors of paraffin oil and water-based gel, where the former increased in temperature at the end of the application of CUS (stage 2), followed by a decrease in the following stages. Meanwhile, the second conductor had a behavior similar to that found in the present study. 

It has been documented that, to return to basal temperature, approximately 20 min is needed [14]. In this study, the final temperatures did not return to their baseline values after 15 min of the intervention. This could be because, as the temperature increases, local blood flow also increases, creating heat sinks, which dissipate the temperature away from the treated tissues [16,18].

Moreover, it is important to note that the mechanism of thermoregulation may vary from person to person since it depends on factors such as the time of year, personal characteristics, or socio-demographic factors. For example, Norheim et al. [40] evaluated 260 healthy soldiers of the Norwegian army. In the study, their hands were subjected to a cold stimulus, dividing the participants according to the results into fast, intermediate, and slow thermoregulation. It was found that 90% of the soldiers evaluated had total (fast) thermoregulation after cooling, while the remaining 10% obtained partial (intermediate and slow) thermoregulation. The participants with slow thermoregulation correlated with a low average hand temperature before the cooling test.

Piva et al. [19] mentioned that thermography can measure the thermal effects of any intervention in a fast way, ensuring reliability and repeatability. IT measures the heat radiated from a distance, without the need for physical contact, generating information on physiological adjustments, imbalances, and thermal responses to physiotherapy treatment. In addition, the thermogram allows the identification of the hottest points of the object, and the exact temperature value of each of them. It verifies the uniformity of the temperature using a color palette [21].

IT has several advantages, including being objective, fast, and non-invasive [27,28], compared with the intramuscular thermometer technique, which can generate risks such as infections, neurogenic inflammation, and skin lesions. In addition, the area of application of the intramuscular thermometer can alter the recording of the temperature rise [41]. It has been proven that the introduction of a needle into the tissue activates the blood and lymphatic microcirculation, causing local vasodilatation, edema, and release of ATP and substance P [42,43] factors that trigger inflammation, resulting in flushing, swelling, pain, and heat, i.e., an increase in temperature [1]. Therefore, invasive techniques can alter the results of the final temperatures recorded. In addition, Liceralde [41] mentions that the temperature was higher when placing the thermocouple in the center of the treatment area than when placing the thermocouple in the periphery. This factor is not considered in some of the studies.

Finally, some limitations of this study should be noted. For this research, we worked only with healthy patients; however, it would be desirable to carry out a study with patients with some alterations to evaluate if, with the use of thermography and ultrasound, it is possible to detect any alterations. Another limitation of the study was that it was performed only on young people, which means that the results cannot be generalized to all ages. Therefore, broadening the age range would be useful to identify possible thermographic changes between different age groups. In addition, an analysis by sex was not performed, due to the lack of data in each group; therefore, it is proposed that it be considered in future studies. It is also proposed to extend the time of measurement to provide long-term follow-up of the thermal effects of CUS. Moreover, gel was used as a coupling medium; in further research, other coupling media could be evaluated together. Finally, the region of interest was selected manually, so an automatic selection would be necessary.

## 5. Conclusions

Therapeutic ultrasound is one of the most widely used tools in physiotherapy; however, there is much controversy about its action and efficacy in treatment. One of the main causes of this controversy is that several studies have described effects like the placebo effect. However, in this study, where the thermal effects were evaluated, two statistically significant variables showed the opposite: the physiological mechanism and the quantitative difference in the decrease and increase in temperature in each dosage.

Therefore, this study demonstrates that the dose of CUS at 3 MHz at 1 W/cm^2^ applied for 5 min achieves thermal effects on muscle tissue in contrast to the application of 3 MHz at 0.5 W/cm^2^. This leads us to consider the clinical applications of this study. Having demonstrated that CUS (3 MHz at 1 W/cm^2^) does have thermal effects compared with a placebo, the authors recommend its use. However, in future research it is suggested to analyze doses equal to or lower than 0.5 W/cm^2^ for 10 min, since in this research no significant results were obtained.

Additionally, the dosage of CUS at 3 MHz at 1 W/cm^2^ applied for 5 min maintained a temperature increase for 15 min, which means that during that time there is a therapeutic effect, that is, the tissue repair process would be carried out, which would mean improvement in the functional capacity of the patient in terms of pain and readiness for movement.

Therefore, this study establishes a guideline on the importance of knowing the thermal effect at different dosages, emphasizing that the key to obtaining its thermal benefits is adequate use at different intensities. However, it is suggested that new studies consider the parameter between 0.6 and 0.9 W/cm^2^, allowing us to determine the minimum intensity to generate a therapeutic thermal effect.

It is essential to highlight the importance of determining the area of application related to the size of the head to consider the rhythmic displacement of the head using a specific technique of passes and specific dosage according to the desired effects. Moreover, the quantity of gel should be quantified, since in this study 2 mL per 50 cm^2^ was used. These two factors are important, as they can greatly influence the period of temperature decrease.

Furthermore, thermography is highlighted as a new tool for diagnostic processes or verification of the effectiveness of some physiotherapy treatments. It stands out for being a non-invasive method, without risk to the health and integrity of the patient. Thus, this study emphasizes the need to continue with more studies to evaluate the thermal effects of physical agents commonly used in clinical practice, such as hot wet compresses and the application of ice, laser, and infrared light, in order to establish specific evidence-based dosing guidelines. 

## Figures and Tables

**Table 1:** Statistical analysis of Group 1—ultrasound at $1\;W/{cm}^{2}$—ultrasound at 1 W/cm^2^.

ROI1	ROI2	${\bar{X}}_{1}$	${\bar{X}}_{2}$	p	$\sigma_{1}$	$\sigma_{2}$	∆T	d
Left Leg 1	Left Leg 2	30.85	26.75	<0.001	1.15	1.62	−4.10	3.71
Left Leg 2	Left Leg 3	26.75	29.04	<0.001	1.62	1.16	2.29	3.18
Left Leg 3	Left Leg 4	29.04	29.61	<0.001	1.16	1.09	0.57	1.66
Left Leg 4	Left Leg 5	29.61	29.79	<0.001	1.09	1.03	0.18	1.37
Left Leg 1	Left Leg 5	30.85	29.79	<0.001	1.15	1.03	−1.06	3.18

**Table 2:** Statistical analysis of Group 2—ultrasound at $0.5\;W/{cm}^{2}$—ultrasound at 0.5 W/cm^2^.

ROI1	ROI2	${\bar{X}}_{1}$	${\bar{X}}_{2}$	p	$\sigma_{1}$	$\sigma_{2}$	∆T	d
Left Leg 1	Left Leg 2	30.79	26.52	<0.001	1.00	1.93	−4.27	2.75
Left Leg 2	Left Leg 3	26.52	28.59	<0.001	1.93	1.12	2.07	2.06
Left Leg 3	Left Leg 4	28.59	29.23	<0.001	1.12	0.86	0.64	1.21
Left Leg 4	Left Leg 5	29.23	29.44	<0.001	0.86	0.82	0.21	1.03
Left Leg 1	Left Leg 5	30.79	29.44	<0.001	1.00	0.82	−1.35	2.01

**Table 3:** Statistical analysis of Group 3—placebo.

ROI1	ROI2	${\bar{X}}_{1}$	${\bar{X}}_{2}$	p	$\sigma_{1}$	$\sigma_{2}$	∆T	d
Left Leg 1	Left Leg 2	30.45	25.17	<0.001	1.25	1.84	−5.28	3.82
Left Leg 2	Left Leg 3	25.17	27.89	<0.001	1.83	1.25	2.72	2.93
Left Leg 3	Left Leg 4	27.89	28.74	<0.001	1.25	1.09	0.85	3.00
Left Leg 4	Left Leg 5	28.74	29.06	<0.001	1.08	1.02	0.32	1.35
Left Leg 1	Left Leg 5	30.45	29.06	<0.001	1.25	1.02	−1.39	2.25

## Data Availability

Not applicable.
